# Do staff capacity and performance-based budgeting improve organisational performance? Empirical evidence from Chinese public universities

**DOI:** 10.1057/s41599-023-01523-2

**Published:** 2023-01-21

**Authors:** Liying He, Kamisah Ismail

**Affiliations:** Department of Accounting, Faculty of Business and Economics, University of Malaya, Kuala Lumpur, Malaysia

**Keywords:** Business and management, Education

## Abstract

The COVID-19 pandemic has raised many issues for higher education institutions, one of which is the continued decline in funding and an increased emphasis on effectiveness and efficiency. Performance-based budgeting is being adopted in organisations to allocate resources more efficiently, and Chinese public universities are no exception. The present study explicitly aimed to examine the relationship among staff capacity, performance-based budgeting, and organisational performance in Chinese public universities. It also investigated the role of top management support as a moderator. A purposive sampling method was used to select a total of 271 participants who agreed to participate in an online survey. A multimethod approach combining partial least squares-structural equation modelling (PLS-SEM), the PROCESS macro and necessary condition analysis (NCA) was adopted. The PLS-SEM results indicated that performance-based budgeting had a positive relationship with university performance and served as a mediator between staff capacity and university performance. The moderated mediation results showed that top management moderated the relationship among the selected variables. The NCA results suggested that both staff capacity and performance-based budgeting are meaningful and significant necessary conditions for university performance. The combined results indicated how researchers and practitioners can identify the factors that are critical for university performance and result in the best possible outcomes. This is possibly the first study to use this multimethod approach in accounting research. Overall, this study offers valuable insights into performance-based budgeting implementation in higher education institutions and may serve as a guideline for public universities to improve the efficiency of funding, reduce costs and increase revenues.

## Introduction

New public management (NPM) has led to critical changes in higher education, requiring universities to focus more on effectiveness and efficiency, resulting in profound changes (Hagerer, 2019). The public’s choice of higher education institutions is based on the nature (quality and qualifications) of the institution and the enrolment scale, which has led to new requirements for developing higher education institutions (Jha, 2021). Universities have experienced financial challenges during the COVID-19 pandemic and must fully consider performance issues to do the most with less money. Thus, universities have introduced many business practices to improve efficiency, increase accountability and enhance budget transparency (Aldhobaib, 2022; Wang and Jones, 2020). Performance-based budgeting (PBB) is adopted in organisations to allocate resources more efficiently. PBB has attracted substantial attention in public management research in recent years (Hyndman et al., 2014; Mauro et al., 2017). PBB is built on the systematic use of performance information to strengthen the link between funds and results. The traditional budget method focuses on input, whereas PBB focuses on output. Previous studies (Aliabadi et al., 2019; de Vries et al., 2019; Jongbloed and Vossensteyn, 2001; Pratolo et al., 2020; Sangiumvibool and Chonglerttham, 2016) that addressed PBB in higher education called for more research in countries other than the United States. This study responds to this call by studying PBB in Chinese public universities.

In China, PBB has been implemented for only slightly more than ten years. Most organisations implement PBB solely for a particular project or only for some budgets. Chinese public universities differ in some ways from other international world-class universities. The most important feature of Chinese public universities is the tradition of bureaucracy with a highly centralised management system. Power in China is an essential factor affecting many reforms. Nuhu et al. (2016) called for further studies on the effect of top management support. Few studies have examined the moderating effect of top management support (Ali et al., 2020; Hsu et al., 2019; Islam et al., 2009). These studies were neither about PBB nor in the field of higher education. This research gap should be addressed in future research.

Furthermore, it has been reported that by 2022, there would be a large number of universities in China, totalling 3013. The main goal of Chinese public universities is to “build a world-class university” (Zhuang and Liu, 2020). To meet this goal, most top Chinese public universities, such as those in Peking and Tsinghua, have implemented a tenure-track system to evaluate the performance of academic staff (Wang and Jones, 2020). However, accounting staff positions are ‘iron rice bowls’ and are allotted to universities by the government at a certain proportion of the number of university faculty members, called ‘bianzhi’ (Qiao, 2011). Under a lifetime employment system, accounting staff need motivation to accept changes and are reluctant to improve their abilities to adapt to new circumstances. Staff capacity is an essential factor hindering business practice implementation in the public sector (Haldma and Lääts, 2002). Previous research (Amirkhani et al., 2019; Douglas, 2000; Egan and Tweedie, 2018; López and Hiebl, 2015; Melkers and Willoughby, 2001; McNab and Melese, 2003; Schick, 2007) has highlighted the importance of staff capacity for innovative practices. However, there have been few studies of staff capacity in higher education institutions, especially in Chinese public universities. The relationship between staff capacity and PBB is supported only by descriptive research (Amirkhani et al., 2019; Douglas, 2000; Schick, 2007, 2014). Therefore, the motivation for this study was to fill these gaps and provide empirical evidence on the causal relationship among staff capacity, PBB, and university performance in different cultural contexts.

### The objectives and significance of the study

This study aims to fill the research gaps in understanding the influence of staff capacity in Chinese public universities. To address the gaps mentioned above, this study adopts a theory integration approach by combining contingency theory and resource dependency theory. This approach offers a theoretical foundation to examine the role of staff capacity through PBB implementation in Chinese public universities. It also explores the effect of staff capacity on business practices and organisational performance in a lifetime employment system context. To achieve the above objectives, this study addresses the following questions: Does staff capacity affect the implementation of PBB? Does staff capacity affect organisational performance? Does PBB mediate the relationship between staff capacity and organisational performance? Does top management support exert a moderating effect?

To answer the research questions, this study relied mainly on partial least squares-structural equation modelling (PLS-SEM) and necessary condition analysis (NCA) to analyse data from the survey questionnaire. This study contributes to the literature in three ways. First, it is possibly the first study to combine PLS and NCA in accounting research. This joint application of PLS and NCA has the potential to advance theory development and generate actionable implications for research and business practice (Richter et al., 2020; p. 2245). Second, prior studies on PBB have been mainly descriptive and explanatory, with no theoretical basis (Carrillo et al., 2021; Lu et al., 2015). This study fills the gap by combining contingency theory and RDT to extend the theoretical framework. Third, this study contributes to the management literature by verifying the effect of power on organisational performance in testing the moderating role of top management support in the higher education sector. Fourth, it addresses the gap in the literature regarding the scope of the moderation effect assessed by the Johnson–Neyman technique. This study also offers insights for practitioners to improve the efficiency of university funding through PBB, reducing costs and increasing revenues.

## Literature review

### Conceptual clarification

In organisations, one crucial issue that may limit innovation adoption is the abilities and competencies of the available human resources. Previous studies have highlighted the importance of qualified staff as a critical factor associated with the adoption and development of innovations (Amirkhani et al., 2019). López and Hiebl (2015) stated that accounting staff must have multiple responsibilities and roles rather than simply focusing on one managerial area. This study combines the actual situation of Chinese public universities and previous research and defines staff capacity as having the necessary professional knowledge and skills and receiving adequate training. According to Willoughby (2011), ‘Performance-based budgeting is a process for developing and incorporating measurements of the performance of government operations, services, and programs into the budget process, which is intended to introduce some rationality into a traditionally subjective and political decision-making process’ (p. 352). PBB links input cost and performance. In other words, it evaluates an organisation’s results through various forms and combines them with performance. Hsu et al. (2019) defined top management support (TMS) as arranging appropriate resources and support for the success of projects, providing clear direction and commitment to help the company eliminate uncertainties and rationally organise technical resources and capabilities, encouraging necessary innovation activities and clear goals and finally developing innovation goals for the budget. Richard et al. (2009) stated that ‘organisational performance is the ultimate dependent variable of interest for researchers concerned with just about any area of management’ (p. 719). According to previous research (Anderson, 2006; Guthrie and Neumann, 2007; Parker, 2011; Sharifabadi, 2012), university performance can be divided into financial and nonfinancial performance. Nonfinancial performance generally includes research performance and teaching performance.

### Theoretical background

This study combines contingency theory and resource dependency theory (RDT) as its theoretical bases. Contingency theory has a long history in accounting research (Chenhall, 2003; Franco-Santos et al., 2012). The core idea of contingency theory is to determine which organisational forms are most suitable for a specific situation. Contingency theory shows that universal applications are inappropriate. Each organisation has its own characteristics. Contingency theory explains how to design an appropriate accounting system to match the external environment, strategy, organisational structure, and technological development. Regarding organisational performance, organisations can modify, adjust, and deploy business practices according to their characteristics and priorities. In addition, various methods can be used to improve organisational performance. The core point of contingency theory is that there is no universally appropriate method. The choice of practices and the degree of application are based on the actual situation of the organisation (Amirkhani et al., 2019). Contingency theory can link the internal and external contextual variables, PBB and organisational performance, and explore the relationship among them. Thus, this study adopts contingency theory as the theoretical basis.

RDT is one of the most influential theories in organisation theory. As Pfeffer and Salancik (1978) stated, ‘To understand the behaviours of an organisation, you must understand the context of the behaviour, that is, the ecology of the organisation’ (p. 1). Resource dependency is an essential but often overlooked dimension that can help explain managerial and organisational behaviours (Birdsall, 2018). The emphasis on power and clear expression of the strategies available to the organisation are hallmarks of RDT, distinguishing it from other theories (Davis and Cobb, 2010). The main point of RDT is that control over the allocation of resources is an essential source of power in organisations (Kholmuminov et al., 2018). Based on this theory, what happens in an organisation depends on the environment and the contingencies and constraints of that environment, such as resource providers’ requirements (Brusca et al., 2019). A few previous studies (Coupet, 2013; Fowles, 2013; Kholmuminov et al., 2018; Pilbeam, 2012) examined the application of RDT in higher education. The state allocates resources through budgets, and universities control activities through budgets (Anessi-Pessina et al., 2016). The present study also supports the basic idea of RDT by testing whether power affects the budget system and organisational performance.

### Hypothesis development

The logic linking staff capacity and PBB in the theoretical framework is based mainly on Haldma and Lääts (2002). They argued that the shortage of qualified accountants affected the adoption of business practices and called for in-depth research on qualified staff (Haldma and Lääts, 2002). From the perspective of contingency theory, staff capacity is an internal factor. From the perspective of RDT, staff capacity is human resources and influences an organisation’s activities. The current implementation of PBB in universities depends largely on insufficient human resources. According to Zha and Liu (2019), Yan et al. (2019), Qiao (2018) and Chen et al. (2018), the main problem in Chinese universities is the small number of full-time accountants in the finance department and the complicated nature of daily business. University accountants are currently performing simple bookkeeping instead of management work. These factors hinder the implementation of PBB (Hu and Li, 2019). Therefore, studying the relationship between staff capacity and PBB is necessary.

Previous studies (Amirkhani et al., 2019; Douglas, 2000; Egan and Tweedie, 2018; López and Hiebl, 2015; Melkers and Willoughby, 2001; McNab and Melese, 2003; Schick, 2007) on the effect of staff capacity on PBB have had contradictory results. López and Hiebl (2015) stated that accounting staff must have multiple responsibilities and roles rather than focusing on one managerial area. Douglas (2000) stated that accounting staff should also have enough advanced knowledge training. Melkers and Willoughby (2001) focused on the need for adequate resources (time, money, and personnel) to implement reform efforts. If staff capacity is lacking, reforms will fail. McNab and Melese (2003) emphasised the necessary investment in staff capacity. Schick (2007) stated that sufficient expertise is essential to implementing PBB. Egan and Tweedie (2018) concluded that accountants need sustainability training to adopt innovations. In contrast, Prihastiwi and Sholihin (2018) found that the qualifications of internal accounting staff negatively influence the adoption of business practices.

Based on the above discussion, this study postulates that staff capacity positively affects the adoption of PBB. Universities with enough qualified staff will have a deeper understanding of PBB and be more likely to accept PBB. They will have time and energy to implement PBB and then achieve the goals of saving costs and improving efficiency and effectiveness. Universities that lack qualified staff will face considerable obstacles in adopting and implementing PBB. This study tests this proposition through the following hypothesis:

H1: A positive relationship exists between staff capacity and PBB in Chinese public universities.

### Staff capacity and university performance

University performance has been a topic of great concern in recent years. Previous research has shown that university performance evaluations are fiercely competitive. As the output of universities differs from that of the private sector, measurement is ambiguous. Differences between academic and administrative staff are significant, and confidence in the validity and accuracy of assessment needs to be improved; thus, the results of university performance assessments are somewhat complicated (Alach, 2017). Many kinds of studies of university performance exist, but most studies have analysed only university performance measurement systems or indicators. The literature on the factors affecting university performance and how to improve university performance is relatively scant (Heinicke and Guenther, 2019).

The Chinese government has issued relevant documents to promote performance reform in universities. As Qiao (2018) stated, universities are required to strengthen capital investment performance, innovate financial support methods, and highlight performance-oriented capital investment methods with incentive and restraint mechanisms. According to traditional Chinese culture, a project needs three factors to be successfully implemented: human resources, material resources and financial resources, all of which are indispensable (Qiao, 2018). As part of human resources, staff capacity significantly affects organisational performance. Qualified staff can integrate financial and business activities and play an essential role in organisational planning, decision-making, control and evaluation. Eligible accounting staff can help an organisation cope with the constantly changing external environment and increasing financial pressures, better allocate resources and improve organisational performance. Based on the above discussion, this study postulates that staff capacity positively affects university performance. It tests this proposition through the following hypothesis:

H2: A positive relationship exists between staff capacity and university performance in Chinese public universities.

### PBB and university performance

PBB may provide enhanced information that improves decision-making and reinforces the improved achievement of organisational goals; consequently, PBB and useful information elevate organisational performance (Chenhall, 2003). Despite the many kinds of studies of university performance, most research has been conducted only on university performance measurement systems or performance measurement indicators. The literature on the factors affecting university performance and how to improve university performance needs to be updated. The relationship between business practices and university performance needs further discussion. De Vries et al. (2019) found that PBB has different effects in American universities; some are positive, whereas others are negative. Habiburrochman and Rizki (2020) and Pratolo et al. (2020) studied Indonesian universities. The former found that PBB does not affect university performance, whereas the latter found that it has a positive effect.

In China, the budget is an essential political medium (bargaining and allocation of power and resources), governance and management tool (planning and decision-making, guidance and control) and government accountability channel (budget transparency and budget execution). For these reasons, budget and performance evaluations are often linked together. Universities that perform well receive more government funding; for those that do not, their budgets are reduced. In this case, PBB is inseparable from university performance. Given the actual situation of Chinese universities, this study postulates that PBB significantly positively affects university performance. This study tests this proposition through the following hypothesis:

H3: A positive relationship exists between PBB and university performance in Chinese public universities.

### Mediating role of PBB

Organisations have unique characteristics and adopt different budget practices. To explain the accounting practices of each organisation, researchers must look within them (Youssef et al., 2020). Pratolo et al. (2020) stated that PBB mediates between contextual factors and university quality. The logic of treating PBB as a mediating variable is based on contingency theory. The current economic and fiscal crisis has put tremendous pressure on universities, requiring them to cut expenses and ensure a balanced budget while actively supporting economic growth. In other words, the adoption of PBB depends on whether it can increase revenue, reduce expenditures, and improve the efficiency of funding. Only when these functions are fully utilised will they positively affect university performance.

Therefore, the issues of cultural, political, economic, and human resources faced by universities will not directly affect their performance. The effect is indirect through various management practices, one of which is PBB. The budgeting system is a tool to assist planning and control management and plays an intermediary role. In other words, the budgeting system is a mechanism that coordinates various departments of the organisation, controls and measures employee performance, motivates employees, and improves communication. It plays a vital role in supporting the quality of universities. In this process, PBB acts as a control mechanism. The budgeting system influences university performance through culture, politics, economics, and human resources. An indirect relationship exists between staff capacity and university performance through implementing PBB. Therefore, based on the above arguments, the following hypothesis is proposed:

H4: PBB mediates the relationship between staff capacity and university performance in Chinese public universities.

### Moderating role of top management support

The logic of treating top management support as a moderating variable in the theoretical framework is based on RDT. In Chinese universities, top managers have absolute power. Top management support is reflected mainly in the allocation of resources. If top management support is lacking, the implementation of PBB may not be successful. By contrast, top management support can replace or compensate for the relative lack of other resources in the organisation and promote the implementation of PBB (Hsu et al., 2019). In Chinese universities, top managers have absolute power and control all resources. Therefore, the effect of top management support on implementing PBB and university performance should be studied.

A few studies have used top management support as a moderating variable (Ali et al., 2020; Hsu et al., 2019; Islam et al., 2009), and not all of them investigated higher education. The present study examines the moderating role of top management support at higher and lower levels. When the attitude towards PBB is sceptical or neutral, the university will be unable to obtain sufficient resources to implement PBB, and the top managers will not communicate and coordinate when they encounter various problems during the implementation. In this case, it is difficult for PBB to succeed, and it will not positively affect university performance. If the level of top management support is high, that is, the attitude towards PBB is positive, sufficient resources will be allocated to implement PBB, and the top managers will actively coordinate the problems and supervise the implementation. PBB can then reduce costs, improve efficiency, and positively affect university performance. Based on the above discussion, this study postulates that top management support moderates the relationship between PBB and university performance. This study tests this proposition through the following hypotheses:

H5: Top management support moderates the relationship among staff capacity, PBB and university performance in Chinese public universities.

H5a: Top management support moderates the relationship between staff capacity and PBB in Chinese public universities.

H5b: Top management support moderates the relationship between staff capacity and university performance in Chinese public universities.

H5c: Top management support moderates the relationship between PBB and university performance in Chinese public universities.

**Fig. 1:**
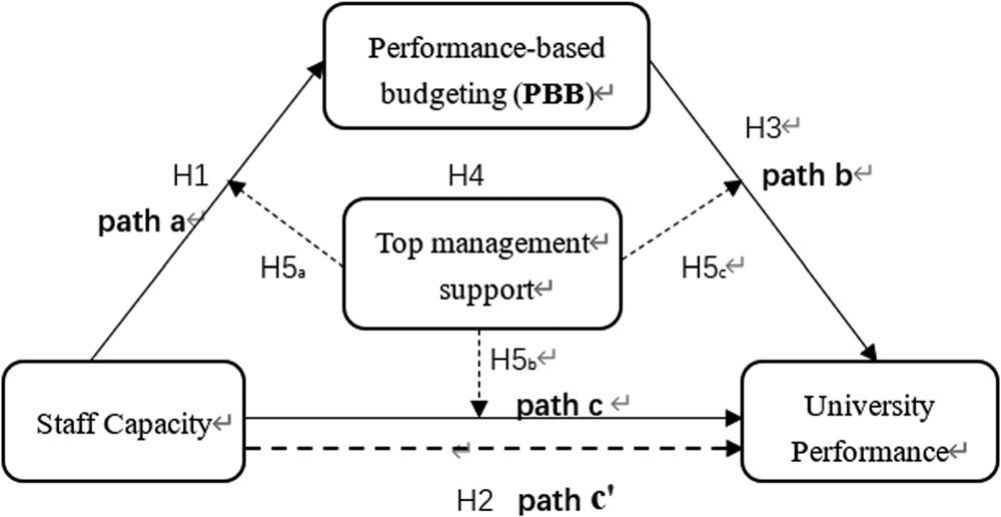
Research model. A description of the relationship between variables in the present study.

In summary, the testing of the five hypotheses in this study is shown in Fig. 1 (research model). Figure 1 shows the path analysis used to test the direct influence of staff capacity on performance-based budgeting (path *a*) and university performance (path *c*), to test the indirect influence of staff capacity on university performance through performance-based budgeting (path *b* and path *c*’) and finally to test the moderating influence of top management support on performance-based budgeting and university performance.

## Methodology

### Research design and participants

The research design guides the progress of a research project and provides relevant information for effectively solving the research problems or hypotheses (Hair et al., 2007). This research focuses mainly on examining the relationship among selected variables in Chinese public universities. Thus, this study employed a quantitative research design and a questionnaire survey to test the proposed hypotheses. The target population was the accounting staff of Chinese public universities’ financial departments as well as the directors of other financial departments, such as the director of the audit department. These people were chosen because not all departments or all employees of the university have an understanding of PBB. Only those in the financial department or audit department with an accounting background can understand PBB and understand the current actual situation of university PBB. The Human Research Ethics Committee approved the questionnaire and methodology for this study.

### Sample and data collection procedure

For this study, purposive sampling was chosen for data collection because it ensured that data were collected only from participants who had relevant knowledge and experience with Chinese public university management. Hair et al. (2019) proposed determining the required sample size, and researchers should use power analyses that take into account the model structure, expected significance level, and expected effect sizes. Based on the effect sizes reported by previous studies, an a priori power analysis was performed using G*Power 3.1. The projected minimum sample size required for a small effect size of f2 = 0.05, with alpha = 0.05 and power = 0.80, is ~*N* = 159.

The questionnaire items were adapted and modified as required from the literature. The questionnaire was sent to five academic scholars in accounting and research methodology areas and five potential respondents in Chinese public universities for a pilot study. Afterwards, the content of the questionnaire was revised in accordance with the suggestions and comments of the experts and potential respondents to improve its clarity and comprehensibility. The final standardised questionnaire was created and distributed through online platforms. In line with Mei and Brown (2017), this study used Wenjuanxing (https://www.wjx.cn), the largest Chinese online survey platform, where the survey was open for four weeks in May 2021 (after receiving approval from the Human Research Ethics Committee). Additionally, WeChat groups and QQ groups (only groups for university accounting staff) were used to collect a sample of the relevant population. Participation was anonymous and completely voluntary, and participants had the choice to withdraw from the survey at any time. After several follow-up rounds, a total of 271 responses were received. No missing data were reported. Since the minimum sample size was 159, the current sample size of 271 participants was suitable for conducting the research.

The sample included 132 male accountants and 139 female accountants. Regarding age composition, nearly half of the respondents (47.6%) were over 45 years of age, 27.3% were 26–35 years of age, and 25.1% were 36–45 years of age. More than half of the respondents (53.1%) had a bachelor’s degree, 36.2% had a master’s degree, and only a few had a doctoral degree (9.2%). Regarding major background, most of the respondents (66.1%) were accounting majors, and the majority of the rest were in majors related to accounting. The results for age, education level and major background indicated that all respondents had the professional knowledge to deal with issues related to PBB in Chinese public universities and could express their views on PBB. Table S1 shows the profile of the respondents.

## Measurement development

Following Churchill’s (1979) guidelines for creating a standardised survey instrument, this study carefully selected the variable measurements based on the research context. All measures were adapted from previous studies and had proven reliability over time. Respondents were asked to rate the situation on a five-point Likert scale ranging from 1 to 5 (1 = strongly disagree, 2 = disagree, 3 = neutral, 4 = agree, 5 = strongly agree). Respondents made corresponding selections based on the actual situation of the university and the degree of compliance with the description. If respondents felt that the actual situation of their university was inconsistent with the description in the questionnaire, they selected 1, and if they felt that the actual situation was completely consistent, they selected 5. The items of the final validated model, along with the descriptive statistics, are listed in Table S2.

### Staff capacity

The measurement of staff capacity was adapted from Amirkhani et al. (2019). Staff capacity had two aspects: number of employees and professional knowledge. The questionnaire contained five items to measure respondents’ views on number of accounting staff and level of qualifications of accounting staff. The reliability of this measure was found to be acceptable with high internal consistency (Cronbach’s alpha = 0.911).

### Performance-based budgeting

PBB links input cost and performance. In other words, it evaluates the organisation’s results through various forms and combines performance with budgeting. Organisations that perform well can obtain more funding, and those that perform poorly experience budget cuts. PBB seeks to improve expenditure efficiency in all its forms by systematically linking funding to results and using performance information to achieve this linkage (de Vries et al., 2019). A questionnaire with ten items developed by Pratolo et al. (2020) was modified to evaluate PBB in Chinese public universities. The reliability of the scale was observed to be 0.881.

### Top management support

Top management support is based mainly on RDT, exploring the impact of power on PBB and university performance during the implementation stage. This study aimed to test the moderation effect of top management support. Top management support was adapted from Islam et al. (2009). This study used top management support as a moderating variable. It included six questions to measure respondents’ views. The reliability of this measure was found to be acceptable with high internal consistency (Cronbach’s alpha = 0.935).

### University performance

In previous studies, countries have had different definitions of key performance indicators and measurement standards. The university performance scale was adapted from the survey instrument developed by Bobe and Kober (2018). The scale was modified (following the feedback on the pilot survey) to suit the Chinese public university management construct as conceptualised in this study. The reliability of the scale was observed to be 0.899.

### Common method bias

As with all self-reported data, common method bias (CMB) can arise from a variety of sources, including the consistency motif and social desirability (Podsakoff et al., 2003; Podsakoff and Organ, 1986). This study used two techniques to reduce the possibility of CMB (Chin et al., 2012; Liang et al., 2007; Podsakoff et al., 2003). First, a Harman single-factor test (Podsakoff and Organ, 1986) was used to test CMB by entering all principal constructs of the theoretical model, including performance-based budgeting, staff capacity, top management support, and university performance, into a principal component analysis (PCA). The results of this test showed that four factors were present, and the highest level of covariance explained by one factor was 38.93 percent, which was lower than the 50% threshold, indicating that the questionnaire was not affected by CMB. Second, following Liang et al. (2007) and Chin et al. (2012), this study used an ad hoc unmeasured latent marker construct (ULMC) approach based on partial least squares-structural equation modelling (PLS-SEM) to detect and control for the influence of common method bias on the analysis. We included all the principal construct indicators and calculated each indicator’s variances substantively explained by the principal construct and by the method. As shown in the additional materials (see Table S3), the results demonstrated that the average substantively explained variance of the indicators was 0.709, while the average method-based variance was 0.060. The ratio of substantive variance to method variance was ~12:1. These results proved that the responses were free of CMB.

### Data analysis

PLS-SEM has been widely used to evaluate complex models (Hair et al., 2018). It is a causal-predictive approach used to develop path models that empirically validate the antecedents that lead to an outcome (Richter et al., 2020). Furthermore, it has the potential to bridge the “dichotomy between explanation, which academic research usually emphasises, and prediction, which is required to derive managerial implications” (Sarstedt et al., 2020, p. 532). This is extremely appealing to both researchers and practitioners seeking to identify the constructs that are important for the development of a specific outcome. Necessary condition analysis (NCA) is a new data analysis technique that enables researchers to look beyond the structural relationships between constructs and identify the necessary conditions in datasets (Dul et al., 2020). Thus, this study employed multiple methods by combining SPSS, PLS-SEM, the PROCESS macro and NCA. SPSS version 26.0 software was used for the descriptive statistical analysis, and Smart PLS and the PROCESS macro software were used for hypothesis testing. Path modelling analysis was then used to examine the proposed hypotheses (H1 to H4) to confirm or reject the relationships between variables. The PROCESS macro available for SPSS (PROCESS version 3.5, model 59) was used to test the moderated mediation hypotheses (H5). The bootstrapping method was used to test the mediation effect. The Johnson–Neyman technique was used to explore the critical value of a significant regression coefficient for the moderating effect of top management support. RStudio software with the R language and NCA package was used for NCA.

## Results

### Descriptive statistics

Table 1 shows the mean, standard deviations, and square root of the average variance extracted from all variables and Pearson’s value correlations (*r*). Based on the table, staff capacity is positively correlated with PBB (*r* = 0.629, *p* < 0.001) and university performance (*r* = 0.425, *p* < 0.001). In addition, PBB is positively correlated with university performance (*r* = 0.737, *p* < 0.001). These results are consistent with our proposed hypotheses and provide preliminary support for them. This study also estimated the variance inflation factors (VIFs) using Smart PLS to check for potential multicollinearity (see Table S4). Each of the variables exhibited a VIF value below the threshold of 3.0 (Picón et al., 2014), ranging from 2.342 to 2.907, with a mean of 2.607, thus indicating that there was no problem with multicollinearity. Based on the benchmark of 0.80 for the strength of the correlations (Franke, 2010), as Table 1 shows, none of the variables was highly correlated. As a result, there were no multicollinearity concerns.

**Table 1:** Descriptive statistics and correlation matrix.

	Mean	SD	SC	TMS	PBB	UNP
SC	3.176	1.032	0.862^a^			
TMS	3.440	0.907	0.737**^b^	0.868		
PBB	3.705	0.697	0.629**	0.629**	0.793	
UNP	3.698	0.661	0.425**	0.588**	0.737**	0.845

*SC* staff capacity, *TMS* top management support, *PBB* performance-based budgeting, *UNP* university performance, *SD* standard deviations.

**Correlation is significant at the 0.001 level (two-tailed).

^a^Square root of the average variance extracted (AVE).

^b^Inter-construct squared correlations.

### Measurement model evaluation

The PLS-SEM assessment initially focused on the measurement models. Examining PLS-SEM estimates enables the researcher to evaluate the reliability and validity of the construct measures (Hair et al., 2018). Hair et al. (2018) suggested that the measurement model assessment should include (1) internal consistency: Cronbach’s alpha (CA) (CA > 0.7) and composite reliability (CR) (CR > 0.7); (2) convergent validity: indicator reliability (factor loadings > 0.7) and average variance extracted (AVE) (AVE > 0.5); and (3) discriminant validity: Fornell–Larcker criterion, cross-loadings, and heterotrait–monotrait ratio (HTMT). According to Hair et al. (2018), the proposed threshold value is HTMT < 0.90 for conceptually similar constructs and HTMT < 0.85 for conceptually different constructs. An HTMT value above 0.85 indicates a lack of discriminant validity (Hair et al., 2018; Nitzl, 2018, 2016). For higher education research, researchers can follow the guiding principles proposed by Ghasemy et al. (2020) to assess the measurement models. This study conducted a one-tailed percentile bootstrapping test (Aguirre-Urreta and Rönkkö, 2018; Ghasemy and Elwood, 2022) with 10,000 subsamples at a 5% significance level to generate confidence intervals for both the reliability and validity measures. Table 2 shows the loadings, convergent validity, and reliability estimates. Table 3 shows the results of the HTMT.

**Table 2:** Loadings, convergent validity, and reliability estimates.

Latent variable	Item	Loading	Alpha	ρ_A	CR	AVE
Staff capacity	SC1	0.880	0.911	0.912	0.935	0.743
	SC2	0.849	**[0.894, 0.926]**	**[0.896, 0.928]**	**[0.923, 0.945]**	**[0.703, 0.775]**
	SC3	0.918				
	SC4	0.930				
	SC5	0.717				
Performance-based budgeting	PBB1	0.788	0.881	0.896	0.910	0.629
	PBB4	0.706	**[0.858, 0.901]**	**[0.877, 0.915]**	**[0.894, 0.924]**	**[0.586, 0.671]**
	PBB5	0.796				
	PBB8	0.729				
	PBB10	0.834				
	PBB11	0.890				
Top management support	TMS1	0.824	0.935	0.936	0.949	0.755
	TMS2	0.895	**[0.918, 0.949]**	**[0.924, 0.951]**	**[0.936, 0.950]**	**[0.709, 0.796]**
	TMS3	0.853				
	TMS4	0.865				
	TMS5	0.891				
	TMS6	0.884				
University performance	UNP2	0.772	0.899	0.905	0.926	0.714
	UNP5	0.867	**[0.875, 0.919]**	**[0.885, 0.925]**	**[0.910, 0.939]**	**[0.669, 0.756]**
	UNP6	0.908				
	UNP7	0.809				
	UNP8	0.862				

*CR* composite reliability, *AVE* average variance extracted; the values in brackets are the lower and upper bounds of the one-tailed percentile confidence intervals with 10,000 subsamples at a 5% significance level.

**Table 3:** Discriminant validity assessment based on HTMT_0.85_ criterion.

Latent variable	PBB	SC	TMS
SC	0.772 **[0.696, 0.841]**		
TMS	0.785 **[0.722, 0.841]**	0.802 **[0.759, 0.841]**	
UNP	0.803 **[0.745, 0.845]**	0.601 **[0.497, 0.699]**	0.596 **[0.526, 0.662]**

*SC* staff capacity, *PBB* performance-based budgeting, *TMS* top management support, *UNP* university Performance; the values in brackets are the lower and upper bounds of the one-tailed percentile confidence intervals with 10,000 subsamples at a 5% significance level.

As shown in Table 2, for internal consistency, the range of Cronbach’s alpha was 0.881 to 0.935. All values were higher than 0.7. The range of composite reliability was 0.910 to 0.949, and all values were higher than 0.7. The lower bounds of the confidence intervals were larger than 0.7, and the upper bounds were desirably smaller than 0.95. The results indicated that the scales were reliable. For convergent validity, the factor loadings were all higher than 0.7. The range of factor loadings was 0.707 to 0.930, the AVE values were all higher than 0.5, the range of AVE values was 0.629 to 0.755, and the lower bounds of the confidence intervals were greater than 0.5. The results indicate that all indicators in the model met the required threshold values of convergent validity. In response to Nitzl’s (2018, 2016) call, HTMT should be used for discriminant validity analysis in future management accounting research. In this study, HTMT was added to analyse discriminative validity. As shown in Table 3, the HTMT values and the upper bounds of the confidence intervals were smaller than 0.85, denoting acceptable levels of discriminant validity according to the HTMT_0.85_ criterion (Franke and Sarstedt, 2019; Hair et al., 2018; Henseler et al., 2015).

### Structural model evaluation

Following Ghasemy et al. (2020), this study used the critical criteria for assessing the structural model in PLS-SEM: coefficients of determination (*R*² values), effect size *f*² and predictive relevance *Q*². This study also used PLSpredict analysis to assess the model’s out-of-sample predictive power (Shmueli et al., 2019). The *R*² and adjusted *R*² of PBB were 0.599 and 0.596, respectively. The *R*² and adjusted *R*² for university performance were 0.536 and 0.531, respectively. These results indicate a moderate level of acceptance. For *f*² effect sizes, the proposed model consisted of one exogenous variable (staff capacity) and two endogenous variables (PBB and university performance). Staff capacity had a large effect on PBB (*ƒ*² = 0.156) and did not affect university performance (*ƒ*² = 0.003). PBB had a large effect on university performance (*ƒ*² = 0.406). For predictive relevance (*Q*²), the *Q*² value of PBB was 0.362 and the *Q*² value of university performance was 0.376. The results showed that all endogenous constructs had medium predictive relevance.

**Fig. 2:**
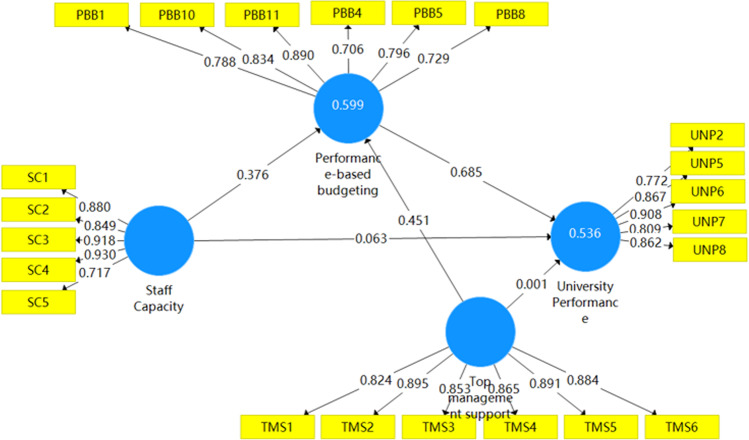
Structural model evaluation. The final model with factor loadings, path coefficients, and endogenous construct *R*^2^.

PLSpredict analysis was used in this study with the default settings (10 folds and 10 repetitions) to evaluate PBB and university performance as the key target constructs. All of the *Q*^2^ predicted values in the PLS results section were above zero. Both the root mean squared error (RMSE) and the mean absolute error (MAE) statistics of ten (of eleven) items in the PLS results section were less than the values for the linear regression model (LM) items. According to Shmueli et al. (2019, p. 2330), if the majority (or the same number) of indicators in the PLS-SEM analysis yields smaller prediction errors than the LM, this indicates medium predictive power. As a result, it was deduced that the proposed model’s out-of-sample predictive performance was medium (Ghasemy et al., 2020; Shmueli et al., 2019). This result is consistent with the results of predictive relevance (*Q*²). Table S5 includes detailed PLSpredict results. Figure 2 displays the final model with factor loadings, path coefficients, and endogenous construct *R*^2^.

### Hypothesis testing

Hypothesis testing is a statistical technique to make decisions to accept or reject hypotheses according to the sample information of the research (Burns and Bush, 2006). Based on the research questions and objectives, this study proposed hypotheses associated with the theoretical model. This study proposed three direct hypotheses, one simple mediational hypothesis (H1–H4) and one moderated mediation hypothesis (H5) to address the research objectives. The main aim of this study was to evaluate the relationship between staff capacity and PBB in Chinese public universities and the effect of PBB on university performance.

#### Direct effect

Table 4 shows the results of evaluating the direct hypotheses. In the research model (see Fig. 1), staff capacity as an independent variable significantly affected performance-based budgeting (path *a*), and staff capacity also significantly affected university performance (path *c*). Performance-based budgeting as a mediating variable significantly affected university performance (path *b*). As shown in Table 4, the results of the direct effect indicate that (1) path *a* indicated that the effect of staff capacity on performance-based budgeting was significantly positive (*β* = 0.712, *t* = 20.502, *p* < 0.001); thus, H1 was supported; (2) path *b* indicated the significant positive influence of performance-based budgeting on university performance (*β* = 0.694, *t* = 10.943, *p* < 0.001), supporting H3; and (3) path *c* indicated that the effect of staff capacity on university performance was nonsignificant (*β* = 0.059, *t* = 0.833, *p* = 0.405 > 0.05). Thus, H2 was not supported.

**Table 4:** Results of the direct effect.

	Relationship	*β*	*T*	*P*	95% CI	Decision
Direct effect test						
H1	SC → PBB	0.712	20.502***	0.000	[0.645, 0.780]	Supported
H2	SC → UNP	0.059	0.833	0.405	[−0.061, 0.213]	Not supported
H3	PBB → UNP	0.694	10.943***	0.000	[0.554, 0.803]	Supported

Standardised estimating of 5000 bootstrap sample, ***p < 0.001, two-tailed.

#### Mediation effect

After direct hypothesis testing, this study investigated the indirect relationship among staff capacity, performance-based budgeting, and university performance. First, the mediation effect was tested. To do so, this study used the method of mediation analysis outlined by Zhao et al. (2010) with recommendations from Rasoolimanesh et al. (2021). The direct effect shows that there is no significant relationship between staff capacity and university performance, in contrast to the traditional Baron and Kenny (1986) approach to mediation analysis. Zhao et al. (2010) argued that a significant direct effect is not required for mediation to occur. This study followed the mediation analysis steps in Rasoolimanesh et al. (2021). The first step was already performed by the direct effect test, and the results showed that the direct effect (path *c*) was nonsignificant. The next step was to investigate whether a significant relationship occurred between the independent variable and the dependent variable by the mediating variable (the indirect effect, path *c*’ = a*b). The last step was using Zhao et al.’s (2010) approach to interpret the signs of indirect and direct effects. Zhao et al. (2010) identified four types of mediation effects: indirect-only mediation, where a mediated effect (a*b) but no direct effect exists; direct-only mediation, where a direct effect (c) but no indirect effect exists; complementary mediation, where a mediated effect (a*b) and direct effect (c) both exist and point in the same direction; and competitive mediation, where a mediated effect (a*b) and direct effect (c) both exist and point in opposite directions (Zhao et al., 2010, p. 200). Table 5 shows the results of the mediation effect. The results indicated significant associations between staff capacity and university performance by performance-based budgeting (*β* = 0.494, *t* = 10.543, *p* < 0.001). The presence of a significant indirect effect but no direct effect in the model suggested that the mediation effect was indirect-only mediation (Zhao et al., 2010). Thus, H4 was supported.

**Table 5:** Results of the mediation effect.

	Relationship	*β*	*T*	*P*	95% CI
Mediation effect test					
Path *a*	SC → PBB	0.712	20.502***	0.000	[0.645, 0.780]
Path *b*	PBB → UNP	0.694	10.943***	0.000	[0.554, 0.803]
Path *c*	SC → UNP	0.059	0.833	0.405	[−0.061, 0.213]
Path *c*'	SC → PBB → UNP	0.494	10.543***	0.000	[0.554, 0.803]

Standardised estimating of 5000 bootstrap sample, ***p < 0.001, two-tail, *c*’ = a*b.

#### Moderated mediation effect

According to Hayes and Rockwood (2020), the moderated mediation effect is called “conditional process analysis”. Conditional process analysis was introduced into the literature by Hayes and Preacher (2013). Although it is a relatively new term, the idea of analytically combining moderation and mediation is not new. Baron and Kenny (1986), James and Brett (1984), and Judd and Kenny (1981) were three seminal articles that combined mediation and moderation effects. Furthermore, in the past decade or so, several important papers and a few books have introduced systematic approaches to integrating moderation and mediation analysis (Becker et al., 2022; Edwards and Lambert, 2007; Fairchild and MacKinnon, 2009; Hayes, 2018; Langfred, 2004; MacKinnon, 2008; Muller et al., 2005; Preacher et al., 2007; Richter et al., 2022; VanderWeele, 2015). This study chose PROCESS (version 3.5) to test H5. A multiple regression model was established by PROCESS (model 59). The results of the regression models are summarised in Table 6.

**Table 6:** Results of the moderated mediation effect.

Predictors	Parametric estimation				95% bootstrapping	
	*β*	SE	*T*	*p*	LLCI	ULCI
Model 1: Outcome variable **Y** = Performance-based budgeting						
Constant	3.655	0.326	11.196	0.000	3.013	4.298
**X** = Staff capacity (SC)	−0.375	0.120	−3.128	0.002	−0.611	−0.139
**Moderator** = Top management support (TMS)	−0.176	0.095	−1.842	0.067	−0.363	0.012
Interaction effect1 (TMS*SC)	0.159	0.029	5.532	0.000	0.102	0.215
*R*^2^	0.715					
Constant	−0.787	0.546	−1.442	0.150	−1.862	0.287
**X** = Staff capacity (SC)	−0.541	0.141	−3.838	0.000	−0.819	−0.264
**Mediator** = Performance-based budgeting (PBB)	1.425	0.204	6.998	0.000	1.024	1.827
**Moderator** = Top management support (TMS)	0.752	0.147	5.121	0.000	0.463	1.041
Interaction effect1 (TMS*SC)	0.112	0.039	2.903	0.004	0.036	0.189
Interaction effect2 (TMS*PBB)	−0.226	0.057	−3.956	0.000	−0.338	−0.113
*R*^2^	0.787					

Bootstrapping results indirect effect	Effect		BootSE		BootLLCI	BootULCI
Conditional indirect effects of staff capacity on university performance						
−1 SD (−0.908)	0.023		0.050		0.072	0.126
*M* (0.000)	0.111		0.033		0.047	0.178
+1 SD (+0.908)	0.140		0.026		0.096	0.199

Model = 59 of Hayes’ PROCESS macro, Bootstrapping samples = 5000; *β* = unstandardised regression coefficient; *SE s*tandard error; *LLCI* lower limit confidence intervals; *ULCI* upper limit confidence intervals. *SD* standard deviation, *M* mean.

Table 6 shows that PBB had a positive significant effect on university performance (*β* = 1.425, *t* = 6.998, *p* < 0.001) and that top management support had a significant positive effect on university performance (*β* = 0.752, *t* = 5.121, *p* < 0.001). Top management support positively moderated the relationship between staff capacity and PBB (TMS * SC, *β* = 0.159, *t* = 5.532, *p* < 0.001) and positively moderated the relationship between staff capacity and university performance (TMS * SC, *β* = 0.112, *t* = 2.903, *p* < 0.01) but negatively moderated the relationship between PBB and university performance (TMS * PBB, *β* = −0.226, *t* = −3.956, *p* < 0.001). These results indicated that top management support moderated the relationship among staff capacity, PBB and university performance. The results also supported H5.

**Fig. 3:**
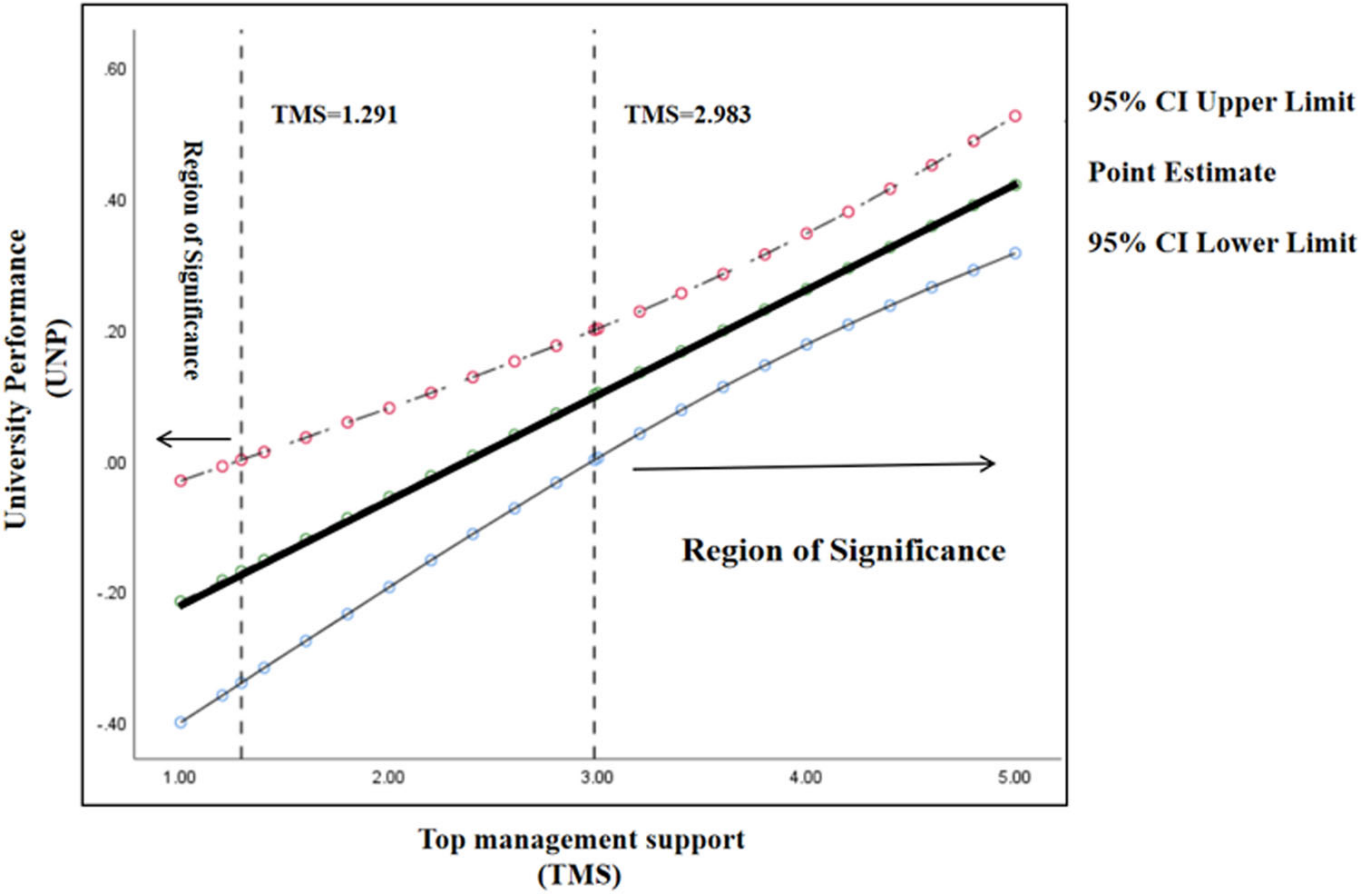
Moderation effect of TMS (Johnson–Neyman analysis). The moderating effect of top management support based on the Johnson-Neyman slope and the region of significance.

In addition, to explain the moderation effect clearly, this study followed the suggestion of Finsaas and Goldstein (2021). It used the Johnson–Neyman technique to identify the range of top management support for which the simple effect of the manipulation was significant. The results revealed that the Johnson–Neyman points were TMS = 1.291 and TMS = 2.983. The moderation effect was significant when top management support was lower than 1.291 and higher than 2.983. The moderation effect was nonsignificant when top management support was between 1.291 and 2.983. Figure 3 shows the Johnson–Neyman slope.

### Robustness checks

To validate the empirical model and research findings, researchers should examine the robustness of several initiatives based on previous studies (Deb et al., 2022). With recommendations from Hair et al. (2019), recent researchers have proposed many complementary methods for assessing the robustness of PLS-SEM results (Hair et al., 2018; Hair et al., 2019; Latan, 2018). These methods addressed robustness testing in either measurement models or structural models. Regarding measurement models, they recommended using confirmatory tetrad analysis (CTA-PLS) to empirically validate measurement model specifications (i.e., reflective versus formative). In terms of the structural model, Sarstedt et al. (2020) suggested that researchers should consider nonlinear effects, endogeneity and unobserved heterogeneity.

For the measurement model, this study applied CTA-PLS, as proposed by Gudergan et al. (2008), to perform a robustness check of the measurement model. According to Hair et al. (2019), if the confidence interval of the measurement model’s tetrad does not include zero, the reflective measurement model should be rejected and a formative measurement model assumed. The additional materials (see Table S6) provide empirical confidence for the reflective mode of the measurement model for constructs. The CTA-PLS results showed that the confidence intervals included zero, which indicated that the model implied-nonredundant tetrads vanished. Therefore, the null hypothesis referring to a reflective measurement model setup was supported.

Next, this study addressed the robustness of the PLS results in terms of detecting the nonlinear relationship of the structural model (Sarstedt et al., 2020). This study did not perform an endogeneity robustness check because its research goal was preliminary prediction. PLS-SEM is causally predictive in the sense that it emphasises prediction in estimating statistical models, the theoretical structures of which are intended to provide causal explanations (Chin et al., 2020). Establishing the predictive power of structural equation models is critical because it ensures their practical utility (Sarstedt and Danks, 2021; Sarstedt et al., 2022). According to Hult et al. (2018, p. 49), if the research goal is preliminary prediction, the researchers can only use the PLS results. If the research goal is the preliminary explanation and endogeneity represents a potential problem, then an endogeneity robustness check should be performed. Based on this benchmark, this study performed only a nonlinear robustness check.

For the nonlinear robustness check, Sarstedt et al. (2020) advised researchers to use Ramsey’s (1969) regression equation specification error test (RESET) to determine whether relationships are nonlinear. They stated that to implement the test in a PLS-SEM context, researchers must first estimate the model and then use the resulting construct scores as input for the RESET, which is available in standard software packages such as Stata and SPSS (Sarstedt et al., 2020). This study followed the method of Sarstedt and Mooi (2019) using SPSS software to perform the RESET. The results showed that neither the regression of staff capacity nor the regression of performance-based budgeting was subject to nonlinearities. Hence, the linear effects model was robust. The results also showed that the change in R2 was not significant, as indicated by the *p*-value of 0.108 (>0.05) under Sig. F Change (see Table S7). These results suggested that the relationships were linear.

As this implementation of the RESET relies on construct scores estimated from a linear effect-only model, researchers should also check whether the specification of a nonlinear effect yields a significant result (Sarstedt et al., 2020). Then, this study created quadratic effects between the constructs using a two-stage approach and assessed their significance using a two-tailed bootstrapping test. The findings revealed that none of the quadratic effects was statistically significant, providing strong support for the proposed model’s robustness (see Table S8 for more details).

### Necessary condition analysis

To further explore the relationship among staff capacity, PBB and university performance, this study supplemented PLS-SEM with necessary condition analysis (NCA). NCA is a new approach and data analysis technique that allows for the identification of necessary conditions in datasets (Dul, 2016). NCA seeks to identify areas in scatter plots of dependent and independent variables that may indicate the presence of a necessary condition rather than analysing average relationships between dependent and independent variables (Richter et al., 2020; Richter et al., 2022). Following the guidelines of Richter et al. (2020), after an assessment of both the measurement and structural models via Smart PLS, this study examined whether staff capacity and performance-based budgeting were necessary conditions for university performance. NCA analysis was carried out using R software. The results of the NCA analysis are displayed in Tables 7 and 8.

**Table 7:** Analysis results of NCA (CE-FDH).

Construct	PBB				UNP			
	Ceiling zone	Scope	Ceiling accuracy (%)	Effect size (*d*) (*p-*value)	Ceiling Zone	Scope	Ceiling accuracy (%)	Effect size (*d*) (*p-*value)
SC	2.633	16.586	100	0.159*** (0.000)	2.558	18.324	100	0.140*** (0.000)
PBB	–				6.198	19.128	100	0.324*** (0.000)

0 ≤ *d* < 0.1 means “low level”; 0.1 ≤ *d* < 0.3 means “medium level”; 0.3 ≤ *d* < 0.5 means “large level”; *d* ≥ 0.5 means “ very large level”. *p*-value represents the permutation test (permutation test, number of redraws = 10,000) in NCA analysis, the closer the *p*-value is to 0, the more significant the impact. ***p < 0.001.

**Table 8:** Bottleneck (in percentages) for university performance.

Bottleneck UNP	SC	PBB
00	NN	NN
10	NN	NN
20	NN	4.7
30	NN	13.1
40	NN	21.4
50	2.2	29.8
60	6.9	38.2
70	11.5	46.6
80	16.2	54.9
90	20.9	63.3
100	25.5	71.7

CR-FDH bottleneck table. *NN* not necessary.

**Fig. 4:**
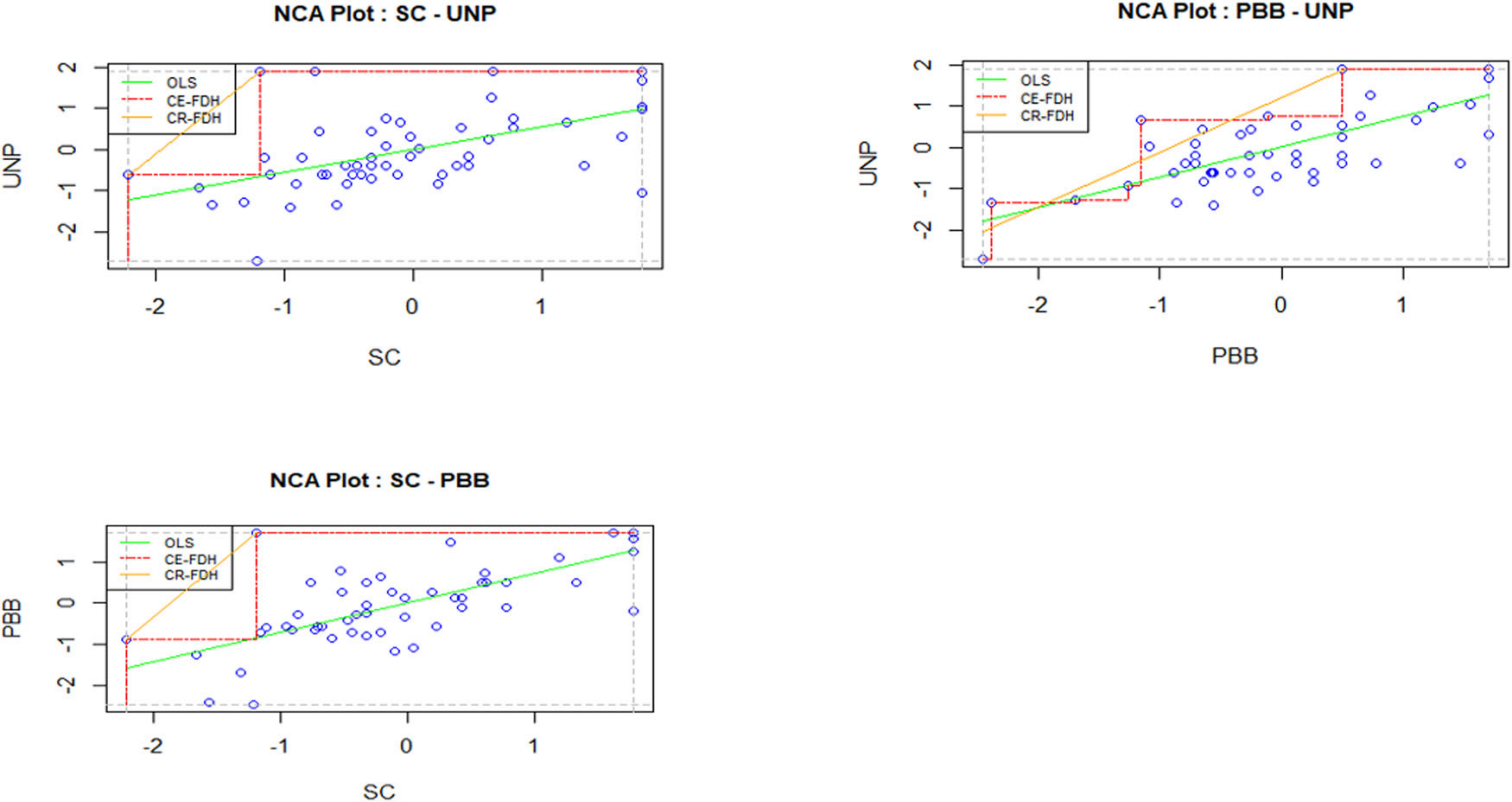
NCA scatter plots. A description of the scatter plots for all relevant relations.

Based on Table 7, the results showed that both staff capacity and performance-based budgeting were meaningful (*d* ≥ 0.1) and significant (*p* < 0.001) necessary conditions for university performance (Richter et al., 2020), with performance-based budgeting being the most necessary condition for university performance (*d* = 0.324, *p* = 0.000). With the bottleneck table, each necessary condition could be evaluated in depth (see Table 8). For example, Table 8 shows that to achieve a 50% level of university performance, two necessary conditions must be met: staff capacity must be at least 2.2%, and performance-based budgeting must be no less than 29.8%. In contrast, for a high level of university performance (100%), staff capacity needs to be at least 25.5%, and performance-based budgeting must be at least 71.7%. This means that if a certain minimum level of staff capacity (25.5%) is not achieved, then the outcome of a high level of university performance will not occur. Figure 4 shows the scatter plots for this effect.

## Discussion and conclusion

### Summary of findings

This study mainly explored the causal relationship among staff capacity, PBB and university performance in Chinese public universities. Combining contingency theory and RDT, we developed a moderated mediation model and empirically examined the proposed hypotheses. The results of PLS-SEM and NCA demonstrated that staff capacity and performance-based budgeting had statistically significant effects on university performance in Chinese public universities. Furthermore, all predictors had varying degrees of necessity and net effects. Table 9 shows the results of this study.

**Table 9:** Overview findings of the study.

Predictor	PLS-SEM results	Hypothesis	NCA results	Interpretation
Staff capacity	Significant	H1: supported	Significant necessary condition	Staff capacity is positively associated with PBB in Chinese public universities. A certain average level of staff capacity is necessary for high implementation of PBB. A further increase in staff capacity will increase the implementation of PBB.
	Nonsignificant determinant	H2: rejected	Significant necessary condition	Staff capacity is not directly associated with university performance in Chinese public universities. But a certain level of staff capacity is necessary for university performance to manifest. However, a further increase is in staff capacity will not directly improved the university performance.
Performance-based budgeting	Significant determinant	H3: supported	Significant necessary condition	A certain average level of performance-based budgeting is necessary for high university performance (see bottleneck table). A further increase in PBB will increase university performance.
	Significant	H4: supported	–	Staff capacity is positively associated with university performance through the mediating role of PBB in Chinese public universities.
Top management support	Significant	H5: supported	–	Top management support moderates the relationship between staff capacity, PBB and university performance in Chinese public universities.

Several interesting findings were revealed from the results of this study. First, the results proved that staff capacity is a necessary condition for PBB and university performance. Staff capacity had a significant positive influence on PBB but not on university performance. The results confirmed the findings of prior studies (Amirkhani et al., 2019; Douglas, 2000; Egan and Tweedie, 2018; López and Hiebl, 2015; Melkers and Willoughby, 2001; McNab and Melese, 2003; Schick, 2007), suggesting that staff capacity is essential to the adoption of innovations. The findings also highlighted the importance of staff capacity in the adoption of PBB. Universities with enough qualified staff will have a deeper understanding of PBB and will be more likely to accept PBB. Second, consistent with previous research conducted in Indonesian universities (Pratolo et al., 2020), our results showed that staff capacity and PBB are both necessary conditions for university performance, but PBB is the most necessary condition, and staff capacity has no direct impact on university performance, which corresponds to the current situation of Chinese public universities. PBB had an indirect-only mediation effect between staff capacity and university performance. The results further verified the reliability and validity of the data we collected.

Furthermore, the research also confirmed resource dependency theory (Pfeffer and Salancik, 2003) and supported the moderation effect of top management support in Chinese public universities. This finding was in accordance with Hsu et al. (2019), who argued that top management support can replace or compensate for the relative lack of other resources in an organisation and promote the implementation of PBB. The most important feature of Chinese public universities is the tradition of bureaucracy with a highly centralised management system. Power in China is an essential factor affecting many reforms. In Chinese universities, top managers have absolute power. If top managers do not support a project, no matter how abundant other resources are, the university cannot implement the project.

### Theoretical implications

The present study enriches the body of knowledge in three significant ways. First, it is possibly the first study to combine PLS and NCA in accounting research. According to Richter et al. (2022), the combination of PLS and NCA is relatively new. There was no study in management research. NCA has already received significant attention in management research (Hauff et al., 2019; Richter et al., 2020; Richter et al., 2022) and has been recommended for the analysis of necessary conditions (Fainshmidt et al., 2020; Richter et al., 2022). PLS is still not a dominant methodology in management accounting and higher education research (Ghasemy et al., 2020; Nitzl, 2018, 2016). This joint application of PLS and NCA has the potential to advance theory development and generate actionable implications for research and business practice (Richter et al., 2020, p. 2245). Previous studies (Lu et al., 2015; Mauro et al., 2017) have either conducted only qualitative research on the factors that influence PBB or have measured only the impact of specific factors on PBB (Pratolo et al., 2020), with none investigating the necessary conditions that influence PBB in depth. Our findings fill this gap and contribute to a better understanding of how staff capacity and PBB influence university performance, thus providing empirical insight for PBB implementation and its impact on university performance in public universities.

Second, this study enriches and expands the literature by combining contingency theory and RDT to develop a novel model to discuss the role of PBB in Chinese public universities. Previous studies on PBB have not applied a unified theory, and more than 90% have had no theoretical basis (Carrillo et al., 2021; Lu et al., 2015). The mature development of PBB needs theoretical support (Carrillo et al., 2021; de Vries et al., 2019; Lu et al., 2015; Mauro et al., 2017). Specifically, the motivation for this study was to respond to the call for research by Mauro et al. (2017), who advocated appropriate theoretical studies in countries other than the United States and proposed combining accounting and management research to further facilitate PBB implementation. Previous research models have applied either only mediation or only moderation models (Pratolo et al., 2020). This study expands the existing theoretical model and adds a moderating variable based on the mediation effect, which changes the theoretical model of this study from a simple mediation model to an integrated conditional process model. The proposed model is established in the context of Chinese public universities. The results are consistent with the actual situation in Chinese public universities.

In addition, this study responds to Nuhu et al.’s (2016) suggestion that the effect of top management support should be studied in the future. However, the moderating effect of top management support has largely been unexamined in the literature (Ali et al., 2020; Hsu et al., 2019; Islam et al., 2009). No previous studies have investigated PBB and higher education. Hence, the current research gap regarding top management support exists because previous studies have focused on whether top management support exists. Therefore, an in-depth study on the degree of top management support needs to be included. This study fills this gap by using top management as a moderating variable, and our findings indicate that the moderated effect is supported.

Finally, this study contributes to accounting research by showing the scope of the moderation effect. Most previous studies in higher education (Mizani et al., 2022) have tested the moderated mediation model. They have all used only the simple-slope two-line analysis (the low level and the high level), but none have showed the scope of the moderation effect. This research used a new data analysis method (the Johnson–Neyman technique) to fill the gaps in the existing management accounting research methods. According to Finsaas and Goldstein (2021), although the simple-slope analysis is instrumental, it also has shortcomings. It ignores some detailed information about the interaction. They stated that the Johnson–Neyman technique could compensate for this shortcoming. The Johnson–Neyman technique is performed to explore the critical value of a significant regression coefficient. The Johnson–Neyman technique is an alternative method for probing concrete interaction terms, but visual depictions of this technique are still in their infancy.

### Practical implications

The practical contribution of the current study is that the results can be used to improve the efficiency of university funding through PBB, reduce costs and increase revenue, thereby improving the internal management level of the university and advancing its core competitiveness. Implementing the new government accounting system, constructing ‘double first-class’ universities and other such policies and institutional factors have provided institutional guarantees for Chinese universities. What Chinese universities need now is the internal motivation to implement PBB. Through this study, universities can become aware of how staff capacity affects PBB and public university performance. This study provides a new perspective and a new method for future research. Future researchers can use our findings to understand the impact of power on university management and can learn from the research methods of this study, expand the scope of research, and conduct related research in combination with the actual situation in Western countries or developing countries.

Another practical contribution of the current study is that it can guide the establishment of management accounting systems, especially PBB, in Chinese provincial universities. Focusing on management accounting in universities can also provide many accountants in China with a way to apply PBB in universities so that the accountants can be freed from tedious daily reimbursement business and have more time and energy to carry out management work.

### Limitations and further research

This study has some limitations that need to be addressed in future research. First, it was conducted in only one country (China), which may limit the generalisability of its findings. Chinese universities are unique compared to other universities, but power exists in any university worldwide. Future researchers can use our findings (with caution) to better understand the impact of power on university management and then explore different cultural settings and their effects on university performance. Second, our analysis tested only one influencing factor (staff capacity). Many other factors, such as national culture, financial pressure, and political influence, can be tested in future research to make our findings more comprehensive and inclusive. Third, staff capacity and university performance were examined only in relation to participants’ attitudes at the same fixed time node, which is known as a cross-sectional study. The cross-sectional research design limited the richness of information available for analysis. As a result, we suggest that future studies use a longitudinal design and data from multiple sources to explore the causality of the relationship. Finally, as evidenced by the numerous extensions and controversial debates on PLS-SEM in recent years, PLS analysis research has become highly dynamic in an effort to compensate for its limitations. This rapid progress has made it difficult for researchers to keep up with the latest developments and decide which research direction to follow. For example, recent PLS-SEM guides recommend seeking potential sources of endogeneity and performing robustness checks to increase confidence in estimation results and conclusions. Robustness checks of endogeneity and heterogeneity should be performed in future accounting research. Recent research has also presented various developments that could soon play very important roles in future research, such as higher-order constructs, model fit assessment, fsQCA, NCA, and consistent PLS. For future studies, researchers should select their own advanced analysis technique based on the specifics of their research aims in order to enhance the development and analysis capabilities of PLS.

## Supplementary information

Supplemental materials

## Data Availability

The data supporting the study’s findings are available upon request from the author.
