# Supplementary material for: Do staff capacity and performance-based budgeting improve organisational performance? Empirical evidence from Chinese public universities
Source: Humanit Soc Sci Commun. 2023 Jan 21;10(1):29. doi: 10.1057/s41599-023-01523-2 (PMC9862238; doi:10.1057/s41599-023-01523-2)
Supplement: Supplementary file 1 — Supplemental materials [file 41599_2023_1523_MOESM1_ESM.docx]

**Do staff capacity and performance-based budgeting improve organisational performance? Empirical evidence from Chinese public universities**

He Liying & Kamisah Ismail

Department of Accounting, Faculty of Business and Economics, University of Malaya, Kuala Lumpur, Malaysia

**Table S1 Profile of the survey respondents**

| **Demographic Item** | **Categories** | **Frequency** | **Percent** |
| --- | --- | --- | --- |
| **Gender** | male | 132 | 48.7 |
|  | female | 139 | 51.3 |
| Total | | 271 | 100.0 |
| **Age** | 26-35 | 74 | 27.3 |
|  | 36-45 | 68 | 25.1 |
|  | Above 45 | 129 | 47.6 |
| Total | | 271 | 100.0 |
| **Education** | Bachelor | 144 | 53.1 |
|  | Master | 98 | 36.2 |
|  | Doctoral | 25 | 9.2 |
|  | Others | 4 | 1.5 |
| Total | | 271 | 100.0 |
| **Major** | Accounting | 179 | 66.1 |
|  | Business administration | 20 | 7.4 |
|  | Economics | 35 | 12.9 |
|  | Finance | 27 | 9.9 |
|  | Others | 10 | 3.7 |
| Total | | 271 | 100.0 |
| **Position** | Financial accountant | 159 | 58.7 |
|  | Internal auditor | 5 | 1.8 |
|  | Director of Finance Department | 60 | 22.1 |
|  | Director of Audit Department | 13 | 4.8 |
|  | Vice President of Finance | 14 | 5.2 |
|  | Others | 20 | 7.4 |
| Total | | 271 | 100.0 |
| **Experience** | Less than 1 year | 5 | 1.8 |
|  | 2 to 5 years | 19 | 7.1 |
|  | 6 to 10 years | 73 | 26.9 |
|  | Above 10 years | 174 | 64.2 |
| Total | | 271 | 100.0 |
| **Qualification** | CPA | 30 | 11.1 |
|  | CIMA | 4 | 1.5 |
|  | CTA | 5 | 1.8 |
|  | Others | 232 | 85.6 |
| Total | | 271 | 100.0 |
| **Professional** | Assistant Accountant | 10 | 3.7 |
| **technical title** | Intermediate Accountant | 104 | 38.4 |
|  | Senior Accountant | 89 | 32.8 |
|  | Intermediate Economist | 15 | 5.5 |
|  | Senior Economist | 29 | 10.7 |
|  | Others | 24 | 8.9 |
| Total | | 271 | 100.0 |

| **Code** | **Item** | **Mean** | **SD** |
| --- | --- | --- | --- |
| SC1 | Number of staff with finance or accounting academic qualifications is adequate | 2.782 | 1.427 |
| SC2 | Our university give all accounting staff to enough training for improve their knowledge | 3.288 | 1.119 |
| SC3 | Accounting staff have sufficient professional knowledge about performance-based budgeting | 3.030 | 1.171 |
| SC4 | Accounting staff know how to implement performance-based budgeting | 3.085 | 1.189 |
| SC5 | Universities are willing to invest in improving the professional knowledge of accounting staff | 3.694 | 1.051 |
| PBB1 | Our university management prepares a budget according to quantitative performance targets that are formulated based on the vision, mission, and goals of the University | 3.587 | 0.983 |
| PBB4 | The amount of my university budget for this year is determined based on last year's budget realization with adjustments for inflation | 3.502 | 1.184 |
| PBB5 | My university management prepares a budget based on quantitative performance targets for each activity to be completed | 3.638 | 0.962 |
| PBB8 | My university has a standard price document that was referred to during budgeting process | 3.819 | 0.998 |
| PBB10 | In my university, at the end of the fiscal year, it will evaluate financial performance achievements | 3.542 | 1.019 |
| PBB11 | In my university, at the end of the fiscal year, it will evaluate non-financial performance achievements | 3.506 | 1.090 |
| TMS1 | Sufficient incentives were provided by top managers for the implementation of performance-based budgeting | 3.199 | 1.125 |
| TMS2 | There was sufficient commitment to the implementation of performance-based budgeting by top managers. | 3.646 | 0.960 |
| TMS3 | Sufficient resources in Chinese universities were provided for the implementation of performance-based budgeting | 3.232 | 0.995 |
| TMS4 | Sufficient resources in Chinese universities were provided for the implementation of performance-based budgeting | 3.295 | 1.111 |
| TMS5 | The top managers of universities actively support the implementation of performance-based budgeting | 3.579 | 0.991 |
| TMS6 | Top managers identified the implementation of PBB as a critical priority | 3.686 | 1.080 |
| UNP2 | Class size | 3.185 | 1.064 |
| UNP5 | External course/program surveys carried out by government and other institutions | 3.325 | 1.048 |
| UNP6 | Internal course/program surveys carried out by the university/faculty | 3.336 | 0.957 |
| UNP7 | Faculty and staff members satisfaction | 3.498 | 1.045 |
| UNP8 | The university’s reputation and image | 3.450 | 1.054 |

**Table S2 Descriptive statistics of the items based on the final model**

**Table S3 Common Method Bias Analysis**

| **Construct** | **Indicator** | **Substantive Factor Loading (R1)** | **R1^2^** | **Method Factor Loading (R2)** | **R2^2^** |
| --- | --- | --- | --- | --- | --- |
| Performance-based budgeting | PBB1 | 0.880(***) | 0.774 | 0.431 | 0.186 |
|  | PBB4 | 0.930(***) | 0.865 | -0.191 | 0.036 |
|  | PBB5 | 0.717(***) | 0.514 | 0.29 | 0.084 |
|  | PBB8 | 0.788(***) | 0.621 | -0.518 | 0.268 |
|  | PBB10 | 0.849(***) | 0.721 | -0.058 | 0.003 |
|  | PBB11 | 0.918(***) | 0.843 | 0.016 | 0 |
| Staff capacity | SC1 | 0.834(***) | 0.696 | -0.142 | 0.02 |
|  | SC2 | 0.890(***) | 0.792 | -0.095 | 0.009 |
|  | SC3 | 0.706(***) | 0.498 | -0.163 | 0.027 |
|  | SC4 | 0.796(***) | 0.634 | -0.08 | 0.006 |
|  | SC5 | 0.729(***) | 0.531 | 0.661 | 0.437 |
| Top management support | TMS1 | 0.824(***) | 0.679 | 0.367 | 0.135 |
|  | TMS2 | 0.895(***) | 0.801 | -0.109 | 0.012 |
|  | TMS3 | 0.853(***) | 0.728 | 0.102 | 0.01 |
|  | TMS4 | 0.865(***) | 0.748 | 0.023 | 0.001 |
|  | TMS5 | 0.891(***) | 0.794 | -0.159 | 0.025 |
|  | TMS6 | 0.884(***) | 0.781 | -0.189 | 0.036 |
| University performance | UNP2 | 0.772(***) | 0.596 | 0.08 | 0.006 |
|  | UNP5 | 0.867(***) | 0.752 | -0.067 | 0.004 |
|  | UNP6 | 0.908(***) | 0.824 | 0.074 | 0.005 |
|  | UNP7 | 0.809(***) | 0.654 | 0.006 | 0 |
|  | UNP8 | 0.862(***) | 0.743 | -0.085 | 0.007 |
| **Average** |  | **0.839** | **0.709** | **0.009** | **0.06** |

**Table S4 Collinearity VIFs**

| **Latent variable** | **PBB** | **SC** | **TMS** | **UNP** |
| --- | --- | --- | --- | --- |
| SC | 2.461 | — | — | 2.763 |
| TMS | 2.342 | — | — | 2.907 |
| PBB | — | — | — | 2.564 |

**Note: SC=Staff Capacity, PBB=Performance-based Budgeting, TMS=Top Management support, UNP=University Performance.**

**Table S5 PLSpredict results**

| **Item** | **PLS** | | | **LM** | | | **PLS-LM** | |
| --- | --- | --- | --- | --- | --- | --- | --- | --- |
|  | **RMSE** | **MAE** | **Q²_predict** | **RMSE** | **MAE** | **Q²_predict** | **RMSE** | **MAE** |
| PBB1 | 0.726 | 0.554 | 0.474 | 0.650 | 0.457 | 0.578 | 0.076 | 0.097 |
| PBB4 | 1.134 | 0.887 | 0.130 | 1.342 | 1.008 | -0.217 | -0.208 | -0.121 |
| PBB5 | 0.712 | 0.553 | 0.469 | 0.885 | 0.634 | 0.179 | -0.173 | -0.081 |
| PBB8 | 0.969 | 0.697 | 0.087 | 1.167 | 0.864 | -0.322 | -0.198 | -0.167 |
| PBB10 | 0.867 | 0.631 | 0.311 | 0.973 | 0.706 | 0.133 | -0.106 | -0.075 |
| PBB11 | 0.853 | 0.619 | 0.418 | 1.109 | 0.799 | 0.016 | -0.256 | -0.180 |
| UNP2 | 1.013 | 0.804 | 0.151 | 1.329 | 1.039 | -0.461 | -0.316 | -0.235 |
| UNP5 | 0.965 | 0.715 | 0.177 | 0.996 | 0.781 | 0.122 | -0.031 | -0.066 |
| UNP6 | 0.829 | 0.639 | 0.271 | 0.932 | 0.757 | 0.077 | -0.103 | -0.118 |
| UNP7 | 0.967 | 0.791 | 0.176 | 1.067 | 0.846 | -0.002 | -0.100 | -0.055 |
| UNP8 | 0.959 | 0.779 | 0.207 | 1.132 | 0.913 | -0.105 | -0.173 | -0.134 |

**Note: RMSE=root mean squared error; MAE= mean absolute error; LM= linear regression model**

**Table S6 CTA-PLS test**

| Staff Capacity | Original Sample  (O) | Sample Mean (M) | Standard Deviation (STDEV) | T Statistics (\|O/STDEV\|) | P Values | Bias | CI Low | CI Up | Alpha adj. | Z  (1-alpha) | CI Low adj.**^a^** | CI Up adj. |
| --- | --- | --- | --- | --- | --- | --- | --- | --- | --- | --- | --- | --- |
| 1: SC1, SC2, SC3, SC4 | 0.059 | 0.058 | 0.039 | 1.514 | **0.130** | -0.001 | -0.004 | 0.124 | 0.020 | 2.327 | **-0.030** | **0.151^b^** |
| 2: SC1, SC2, SC4, SC3 | 0.078 | 0.077 | 0.046 | 1.686 | **0.092** | -0.001 | 0.003 | 0.156 | 0.020 | 2.327 | **-0.029** | **0.187^b^** |
| 4: SC1, SC2, SC3, SC5 | -0.070 | -0.069 | 0.044 | 1.596 | **0.111** | 0.001 | -0.143 | 0.001 | 0.020 | 2.327 | **-0.173** | **0.031^b^** |
| 6: SC1, SC3, SC5, SC2 | 0.023 | 0.022 | 0.082 | 0.284 | **0.776** | -0.001 | -0.110 | 0.159 | 0.020 | 2.327 | **-0.166** | **0.215^b^** |
| 10: SC1, SC3, SC4, SC5 | 0.031 | 0.032 | 0.047 | 0.665 | **0.506** | 0.000 | -0.047 | 0.109 | 0.020 | 2.327 | **-0.079** | **0.141^b^** |

**Note: a means adjustment of the 10% bias corrected bootstrap (two-tailed) confidence interval limits uses the Bonferroni method to account for multiple testing issues; b means model-implied nonredundant tetrads that vanish. Standardized estimating of 5,000 bootstrap sample.**

| Performance-based budgeting | Original Sample (O) | Sample Mean (M) | Standard Deviation (STDEV) | T Statistics (\|O/STDEV\|) | P Values | Bias | CI Low | CI Up | Alpha adj. | Z  (1-alpha) | CI Low adj.**^a^** | CI Up adj. |
| --- | --- | --- | --- | --- | --- | --- | --- | --- | --- | --- | --- | --- |
| 1: PBB1, PBB10, PBB11, PBB4 | -0.174 | -0.171 | 0.056 | 3.087 | 0.002 | 0.002 | -0.269 | -0.080 | 0.011 | 2.540 | -0.319 | -0.033 |
| 2: PBB1, PBB10, PBB4, PBB11 | -0.196 | -0.194 | 0.068 | 2.876 | 0.004 | 0.002 | -0.309 | -0.09 | 0.011 | 2.540 | -0.37 | -0.025 |
| 4: PBB1, PBB10, PBB11, PBB5 | -0.016 | -0.015 | 0.015 | 1.064 | **0.287** | 0.000 | -0.040 | 0.008 | 0.011 | 2.540 | **-0.053** | **0.021^b^** |
| 6: PBB1, PBB11, PBB5, PBB10 | -0.405 | -0.401 | 0.06 | 6.814 | 0.000 | 0.005 | -0.508 | -0.310 | 0.011 | 2.540 | -0.561 | -0.259 |
| 7: PBB1, PBB10, PBB11, PBB8 | -0.113 | -0.111 | 0.039 | 2.923 | 0.003 | 0.002 | -0.178 | -0.05 | 0.011 | 2.540 | -0.212 | -0.016 |
| 10: PBB1, PBB10, PBB4, PBB5 | -0.049 | -0.049 | 0.038 | 1.313 | **0.189** | 0.000 | -0.111 | 0.012 | 0.011 | 2.540 | **-0.145** | **0.046^b^** |
| 16: PBB1, PBB10, PBB5, PBB8 | -0.267 | -0.264 | 0.052 | 5.111 | 0.000 | 0.003 | -0.356 | -0.180 | 0.011 | 2.540 | -0.403 | -0.138 |
| 22: PBB1, PBB11, PBB4, PBB8 | 0.03 | 0.029 | 0.062 | 0.480 | **0.631** | -0.001 | -0.072 | 0.132 | 0.011 | 2.540 | **-0.127** | **0.188^b^** |
| 26: PBB1, PBB11, PBB8, PBB5 | -0.036 | -0.036 | 0.023 | 1.576 | **0.115** | 0.000 | -0.075 | 0.001 | 0.011 | 2.540 | **-0.095** | **0.022^b^** |

**Note: a means adjustment of the 10% bias corrected bootstrap (two-tailed) confidence interval limits uses the Bonferroni method to account for multiple testing issues; b means model-implied nonredundant tetrads that vanish. Standardized estimating of 5,000 bootstrap sample.**

| University Performance | Original Sample (O) | Sample Mean (M) | Standard Deviation (STDEV) | T Statistics (\|O/STDEV\|) | P Values | Bias | CI Low | CI Up | Alpha adj. | Z  (1-alpha) | CI Low adj.**^a^** | CI Up adj. |
| --- | --- | --- | --- | --- | --- | --- | --- | --- | --- | --- | --- | --- |
| 1: UNP2, UNP5, UNP6, UNP7 | -0.198 | -0.197 | 0.051 | 3.850 | 0.000 | 0.001 | -0.283 | -0.114 | 0.020 | 2.327 | -0.319 | -0.079 |
| 2: UNP2, UNP5, UNP7, UNP6 | -0.247 | -0.246 | 0.061 | 4.038 | 0.000 | 0.002 | -0.349 | -0.148 | 0.020 | 2.327 | -0.391 | -0.106 |
| 4: UNP2, UNP5, UNP6, UNP8 | -0.117 | -0.116 | 0.038 | 3.051 | 0.002 | 0.000 | -0.180 | -0.054 | 0.020 | 2.327 | -0.206 | -0.028 |
| 6: UNP2, UNP6, UNP8, UNP5 | 0.015 | 0.016 | 0.046 | 0.334 | **0.738** | 0.000 | -0.061 | 0.091 | 0.020 | 2.327 | **-0.093** | **0.123^b^** |
| 10: UNP2, UNP6, UNP7, UNP8 | -0.103 | -0.102 | 0.070 | 1.466 | **0.143** | 0.001 | -0.219 | 0.012 | 0.020 | 2.327 | **-0.267** | **0.060^b^** |

**Note: a means adjustment of the 10% bias corrected bootstrap (two-tailed) confidence interval limits uses the Bonferroni method to account for multiple testing issues; b means model-implied nonredundant tetrads that vanish. Standardized estimating of 5,000 bootstrap sample.**

**Table S7 Model summary ^c^ used for Ramsey’s RESET test**

| **Model** | **R** | **R Square** | **Adjusted R Square** | **Std. Error of the Estimate** | **Change Statistics** | | | | |
| --- | --- | --- | --- | --- | --- | --- | --- | --- | --- |
|  |  |  |  |  | **R Square Change** | **F Change** | **df1** | **df**  **2** | **Sig. F Change** |
| 1 | .518^a^ | .268 | .263 | .74047 | .268 | 49.067 | 2 | 268 | .000 |
| 2 | .524^b^ | .275 | .267 | .73828 | .007 | 2.598 | 1 | 267 | .108 |

**Note：a Predictors: (Constant),** **performance-based budgeting, staff capacity**

**b Predictors: (Constant), performance-based budgeting, staff capacity, PRED_2, PRED_3**

**c Dependent Variable: University performance**

**Table S8 Robustness check: Quadratic effects**

| **Relationship** | **Coefficient** | **95% CI** | ***f^2^*** |
| --- | --- | --- | --- |
| Staff Capacity -> University Performance | 0.101 | [-0.032-0.284] | 0.008 |
| Staff Capacity -> Performance-based budgeting | 0.337(***) | [0.202-0.485] | 0.115 |
| Performance-based budgeting -> University Performance | 0.706(***) | [0.519-0.855] | 0.409 |
| R^2^ - Performance-based budgeting | 0.605 | | |
| R^2^ - University Performance | 0.558 | | |
| Q^2^- Performance-based budgeting | 0.367 | | |
| Q^2^- University Performance | 0.391 | | |
| **Quadratic Effect**  Staff Capacity -> University Performance | -0.108 | [-0.255-0.071] | 0.010 |
| Staff Capacity -> Performance-based budgeting | -0.027 | [-0.157-0.123] | 0.001 |
| Performance-based budgeting -> University Performance | 0.151 | [-0.006-0.364] | 0.026 |

**Notes: *p < .05, **p < .01, ***p <.001; 95% CI Percentile Confidence Interval; Bootstrapping based on n=5000 bootstrap samples; Quadratic effects assessed by applying a two-tailed percentile bootstrap test at 5% of significance level [2.5%-97.5%].**
